# Fahr’s Syndrome for Primary Hypoparathyroidism in a Patient With COVID-19

**DOI:** 10.7759/cureus.26342

**Published:** 2022-06-26

**Authors:** Irene Irisson-Mora, Luis A Rodríguez-Hernández, Juan C. Balcázar-Padrón, Juan Peralta Luzon, Lesly Portocarrero-Ortiz

**Affiliations:** 1 Neuroendocrinology, Instituto Nacional de Neurología y Neurocirugía, Mexico City, MEX; 2 Neurosurgery, Instituto Nacional de Neurología y Neurocirugía, Mexico City, MEX

**Keywords:** calcium deposition, covid-19 pneumonia, calcific lesions, hypo-parathyroidism, fahr´s syndrome

## Abstract

Fahr's syndrome, recently named "primary brain calcification," is a rare disorder with a variable clinical presentation ranging from behavioral changes to seizures. It can be idiopathic or have multiple causes, hypoparathyroidism the most frequent. In the current coronavirus 2019 (COVID-19) pandemic, these electrolyte imbalances have acquired importance, and there has been a correlation between the lowest serum calcium levels and severe COVID-19 disease. It is known that calcium accomplishes many normal physiologic functions.

We present a case of a 63-year-old woman who arrived at the emergency room with a fever of 10-day duration, odynophagia, dry cough, dyspnea, and drowsiness. Upon her arrival, computed tomography of the brain and chest was performed, showing areas of calcification in the basal nuclei and infiltrates with a ground-glass pattern, respectively. In addition, laboratory studies were conducted in which hypocalcemia and hyperphosphatemia stand out. Furthermore, a positive result was obtained from acute Respiratory Syndrome Coronavirus 2 (SARS-COV2) from bronchial secretion. According to the clinical presentation data in the imaging and laboratory studies, Fahr's syndrome and COVID-19 pneumonia were diagnosed.

We consider evaluating electrolyte imbalances at case presentations essential and continuously monitoring them. Appropriate and prompt corrections were achieved in patients with hypoparathyroidism history and severe COVID-19 disease. This case shows the vital collaboration between endocrinologists and other physicians that care for patients with COVID-19 infection.

## Introduction

Fahr's syndrome is a rare neurodegenerative disease with a less than 0.5% prevalence characterized by bilateral symmetric calcifications of the basal ganglia but may also be present in other brain structures. It may be classified as primary or genetic and secondary. Its principal cause is hypoparathyroidism, a disease characterized by low or inappropriately normal parathyroid hormone (PTH) levels in context with hypocalcemia. The clinical characteristics of hypocalcemia in these patients depend on the time and severity of the presentation. Those present in an acute form may compromise the nervous and muscular systems. In contrast, those with a chronic condition may initiate a metabolic cascade that results in ectopic calcifications and ectopic calcium deposits in the brain, which may cause diverse neurological problems [1-3].

The morbidity associated with hypoparathyroidism has been directly related to hypocalcemia and hypophosphatemia. Patients hospitalized for a long time have a higher risk of presenting respiratory tract infections [1,4].

## Case presentation

A 63-year-old woman presented to the emergency room with a persistent fever of 10-day duration, odynophagia, dry cough, dyspnea, and drowsiness. Two days before his arrival, he presented a convulsive crisis characterized by dystonic movements of both hands with flexion of both upper limbs and extension of the lower limbs with loss of alertness lasting one minute associated with loss of sphincter control. The patient has a history of seizures that began in adolescence, characterized by focal motor seizures with a tonic posture on the right side of the body, without altered alertness. In the last year, the number of crises has increased from six to nine per day of 10 seconds duration. Throughout her condition, the patient has received epilepsy treatment with clonazepam, magnesium valproate, and carbamazepine with poor response and eye surgery for bilateral cataracts.

On the physical examination, vital signs were arterial blood pressure of 60/40 mmHg, heart rate of 92, temperature of 36.8°C, breathing rate of 24, oxygen saturation of 78%, chest with intercostal straining, and bilateral crackles. She required advanced airway management and mechanical ventilatory support due to severe hypoxemia. The patient scored nine points on the Glasgow Coma Scale on neurological examination without cranial nerve involvement, sensitivity, or motor examination without alterations. The results of the laboratory carried out can be seen in Table 1.

**Table 1:** Laboratory results *Corrected total calcium (mg/dL) = measured calcium (mg/dL) + 0.8 x [4 – serum albumin (g/dL)]. PTH: parathyroid hormone, 25-OH vitamin D: 25-hydroxyvitamin D.

Parameters	Reference values	Results
Total serum calcium (mg/dL)	8.6-10.2	4
Corrected calcium for serum albumin* (mg/dL)	8.6-10.2	4.8
Phosphorus (mg/dL)	2.7-4.5	8.3
Magnesium (mg/dL)	1.7-2.5	2.1
PTH (pg/dL)	1.2-8.8	2.1
Albumin (g/dL)	3.9-5.1	2.9
25-OH vitamin D (ng/mL)	20-100	31

**Figure 1:**
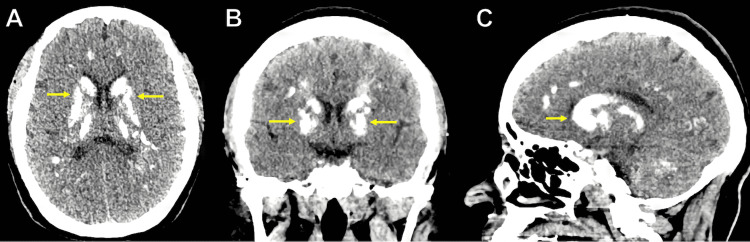
Unenhanced Brain CT scan (A) Axial, (B) coronal, and (C) sagittal reconstructions showing calcified basal ganglia (yellow arrows).

An unenhanced brain computed tomography (CT) scan showed bilateral calcifications in the basal ganglia, thalamus, corona radiata, cerebellar hemispheres, and frontal and parietal lobes (Figure 1A-1C).

**Figure 2:**
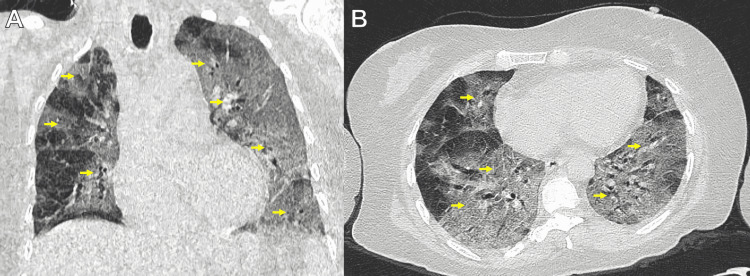
Unenhanced chest CT scan (A) Coronal and (B) axial reconstructions showing bilateral pulmonary infiltrates in all of the pulmonary parenchymas with a ground-glass pattern (yellow arrows).

An unenhanced chest CT scan showed bilateral pulmonary infiltrates in all pulmonary parenchyma with a ground-glass pattern. The COVID-19 Reporting and Data System (CO-RADS) score correspond to the five categories (Figure 2A-2B).

According to clinical presentation biochemical and radiologic results, pneumonia and Fahr's syndrome were diagnosed secondary to primary hypoparathyroidism with severe secondary hypocalcemia and hyperphosphatemia. Management was initiated with calcium gluconate in a continuous infusion, calcium carbonate orally and a phosphorous chelant, resulting in a normalization of the serum calcium concentration. We continued her previous anticonvulsive and antibiotic treatments with vancomycin and meropenem to manage pneumonia. She required vasopressor support due to a hemodynamic collapse associated with septic shock, with a Sequential Organ Failure Assessment (SOFA) score of 18 points.

Due to the radiologic results of pneumonia suggestive of COVID-19, a reverse transcriptase-polymerase chain reaction assay (PCR-RT) for Severe Acute Respiratory Syndrome Coronavirus 2 (SARS-COV2) from bronchial secretion was obtained with a positive result. After three days of hospital stay, she was transferred to the COVID-19 center, where she was managed with hydroxychloroquine and the rest of the previously mentioned treatment. She developed multiple organ failures and death in two weeks posteriorly.

## Discussion

Fahr's syndrome is a rare neurodegenerative disease with a less than 0.5% prevalence characterized by bilateral symmetric calcifications of the basal ganglia but may also be present in other brain structures. It may be classified as primary or genetic and secondary, where its principal cause is hypoparathyroidism, a disease characterized by low or inappropriately normal PTH levels in context with hypocalcemia. The clinical characteristics of hypocalcemia in these patients depend on the time and severity of the presentation. Those that occur acutely can compromise the nervous and muscular systems, causing symptoms such as seizures, cataracts, and altered mental status, such as those presented in this case. In contrast, those in the chronic form may initiate a metabolic cascade that results in ectopic calcifications and ectopic calcium deposits in the brain, which may cause diverse neurological problems [1-3].

Hypoparathyroidism has been associated with a greater risk of comorbidities, including cardiovascular disease, renal insufficiency, neuropsychiatric disorders, seizures, cataracts, intracerebral calcifications, and infections. These complications are directly related to hypocalcemia, hyperphosphatemia, or indirectly due to treatment effects when this is insufficient or excessive. The risk of infections, including respiratory tract infections, is associated with the number of episodes of hypocalcemia, the high phosphate levels, and the disease duration. It is well known that calcium contributes to many primary physiologic functions. Hypocalcemia can affect the immune response to infection because calcium acts as a secondary messenger in neutrophils [4-8].

In the current COVID-19 pandemic caused by the SARS-COV2 virus, many case reports and cohort studies have described multiple clinical characteristics, among which electrolyte alterations stand out. A grouped analysis reported that low serum sodium and potassium chloride concentrations are associated with severe COVID-19 disease. Proposing that patients with severe COVID-19 illness, those who need mechanical ventilation and advanced vital support, tend to show a higher proportion of hypocalcemia at case presentation than those with mild COVID-19 disease. Nonetheless, limited sampling and heterogeneous electrolyte reporting currently have limited interpretations [9-11].

Recently, a higher incidence of multiple organ failure, septic shock, and higher mortality at 28 days was found in patients with COVID-19 and low calcium levels. In addition, hypocalcemia has also been reported in 74.7% of patients with COVID-19 disease [12]. In our case, alterations in calcium metabolism were evident in history and paraclinical studies, such as previous bilateral cataracts and previous seizures. Still, despite the coexistence of severe hypocalcemia in this last presentation, there were no new seizures, suggesting that the speed and duration may have been chronic. Nonetheless, COVID-19 infection has been reported to be a precipitant factor for developing severe hypocalcemia in patients with primary hypoparathyroidism [13].

Her initial symptoms and chest CT scan showed multiple foci of ground glass images, "crazy paving" areas, and some areas with inverse halo perilobular patterns (Figure 2) [14]. There are two similar cases in the literature of association between Fahr's Syndrome and COVID-19. In both cases, the authors report their experience diagnosing this entity and how the disease debuted. In the first case, Demir et al. reported the disease's onset due to tonic-clonic seizures, as in our case. In the second case, Bhangal et al. mentioned that the history of Fahr's Syndrome precipitated the complications of COVID-19 pneumonia due to the metabolic alterations that it causes. In our case, the diagnosis was made by the presence of seizures and laboratory data suggestive of Fahr's syndrome. With this third case, it is evident that the systemic inflammatory response produced by COVID-19 can, in some cases, exacerbate the metabolic alterations that cause Fahr's syndrome and have complications in the evolution of patients [15,16]

The general mortality rate for COVID-19 infection was described as less than 3.6%, adults over 60 years old, patients with a high SOFA score, and patients with several comorbidities, such as our patient, have higher probabilities of developing severe disease associated with higher mortality. Until now, no evidence confirms that patients with COVID-19 and hypoparathyroidism are at risk for higher complications, even though it has been proposed that lower serum calcium levels at presentation may correlate with severe COVID-19 disease [17-20].

## Conclusions

We consider it very important to evaluate electrolyte imbalances and continuously monitor them, and prompt corrections must be achieved in patients with hypoparathyroidism history and severe COVID-19 disease. This case shows the critical collaboration between endocrinologists and other physicians who care for patients with COVID-19 infection, especially in cases with the possibility or history of endocrine diseases, to prevent as many adverse results associated with COVID-19 infection.
